# Hyperinsulinemic Hypoglycemia and Growth Hormone Deficiency Secondary to 20p11 Deletion

**DOI:** 10.1155/2023/8658540

**Published:** 2023-06-26

**Authors:** Erica Wee, John Herriges, Kavitha Dileepan, Sarah L. Tsai, Joseph T. Alaimo, Emily Paprocki

**Affiliations:** ^1^Division of Pediatric Endocrinology and Diabetes, Children's Mercy Kansas City, Kansas City, MO, USA; ^2^University of Missouri-Kansas City School of Medicine, Kansas City, MO, USA; ^3^Department of Pathology and Laboratory Medicine, Children's Mercy Kansas City, Kansas City, MO, USA

## Abstract

Hypoglycemia is concerning for neurological complications in infants and children. Determining the cause of hypoglycemia is essential in providing appropriate treatment. Hyperinsulinism and growth hormone deficiency are known causes of hypoglycemia but are not commonly found together. We report a 4-month-old boy who presented with severe hypoglycemia and was found to have both hyperinsulinism and growth hormone deficiency. Treatment with both recombinant human growth hormone and diazoxide led to blood glucose normalization. Subsequently, he was found to have a genetic diagnosis of 20p11.22p11.21 deletion. 20p11 deletions have been associated with hypopituitarism, most commonly seen in growth hormone deficiency causing hypoglycemia. This case is one of a few to report hyperinsulinism as a manifestation of this deletion.

## 1. Introduction

Hypoglycemia is often the result of a single etiology, but in rare instances, multiple factors can contribute. Hyperinsulinism and growth hormone deficiency are known causes of hypoglycemia but do not commonly present simultaneously. 20p11 deletions have been associated with hypoglycemia due to hypopituitarism, most commonly, growth hormone deficiency [1–5]. Few cases in the literature report the same mutation resulting in hyperinsulinemic hypoglycemia [1–3]. This deletion is also associated with other pituitary hormone deficiencies, including central hypothyroidism, central adrenal insufficiency, and central hypogonadism [2–4, 6]. We report a rare case of a patient with 20p11.22p11.21 deletion who presented with hypoglycemia due to growth hormone deficiency and hyperinsulinism.

## 2. Case Presentation

A 4-month-old, full-term, non-Hispanic white male infant presented with seizures. He was born to a G3P3 mother without history of gestational diabetes and to nonconsanguineous parents. He was born large for gestational age with a birth weight of 4280 grams (96th percentile, SD 1.80) and a birth length of 53.3 cm (96th percentile, SD 1.71). The mother reported that he had transient hypoglycemia requiring dextrose fluids and hyperbilirubinemia requiring phototherapy during the first few days of life. He was discharged home at 5 days old. Newborn screen was normal. The mother had no concerns related to his growth and development. The patient was exclusively breastfed and nursed every 2-3 hours.

He was referred to the Department of Neurology and had a negative 60 hour video EEG. Biochemical evaluation was not completed at that time. He was diagnosed with tonic-clonic seizures based on videos obtained during the event and was started on antiseizure medication, levetiracetam at 30 mg/kg/day. However, as he continued to have breakthrough seizures at home, the dose of levetiracetam was increased to 39 mg/kg/day.

The primary care provider did a biochemical evaluation that was significant for a serum glucose of 54 mg/dL (3 mmol/L) despite the blood being drawn 20 minutes after a breastfeed. This finding prompted admission to the hospital for further evaluation. Examination revealed weight of 6.62 kg (23rd percentile), length of 62.5 cm (15th percentile), and head circumference of 43.5 cm (75th percentile). His midparental height was at the 95th percentile. Physical examination showed no evidence of dysmorphism, macroglossia, hemihypertrophy, or hepatosplenomegaly. He had normal genitalia with normal phallus length and descended bilateral testicles. He had a normal neurological examination. His initial point-of-care blood glucose on admission was 20 mg/dL (1.11 mmol/L) and confirmed with a serum glucose (BG) of 39 mg/dL (2.17 mmol/L). He was asymptomatic and had last fed 2 hours prior to the blood draw. A critical sample revealed detectable insulin, low beta hydroxybutyrate, and low growth hormone (GH). Results of the critical sample are summarized in Table 1. His cortisol response to hypoglycemia was sufficient. A glucagon (0.5 mg) test (Table 2) showed an increase in BG of 26 mg/dL (1.44 mmol/L). He failed a GH stimulation test (Table 3) with arginine (0.5 mg/kg) and glucagon (0.03 mg/kg). He had mildly elevated liver enzymes with ALT 136 (20–77) U/L and AST 56 (≤44) U/L with normal total bilirubin 0.7 (0–1.2) mg/dL. ALT and AST normalized on repeat testing done after 3 months. His electrolytes and kidney function were normal. Plasma amino acids, plasma acylcarnitine profile, and urine organic acids were normal. He had a TSH of 1.48 (0.35–7.6) mcIU/mL and free T4 of 1.1 (0.7–1.9) ng/dL. IGF-1 was 17 (15–189) ng/mL. He had a normal brain and pituitary MRI revealing a normal hypothalamic-pituitary area including a normal neurohypophyseal bright spot demonstrated.

Immediately after completing the test, he was placed on 10% dextrose fluids with a glucose infusion rate (GIR) of 8 to 11 mg/kg/min due to inability to maintain euglycemia on only oral feedings. After the test results were reviewed, he was started on recombinant human growth hormone (rhGH) 0.25 mg daily (equivalent to 0.27 mg/kg/week). The GIR was then decreased to 3-4 mg/kg/min, but he was unable to completely wean off dextrose-containing fluids due to continued hypoglycemia (44–58 mg/dL or 2.44–3.22 mmol/L) when decreasing GIR further. Diazoxide (5 mg/kg/day) and chlorothiazide (10 mg/kg/day) were started after baseline echocardiogram was confirmed to be normal. Diazoxide dose was adjusted over the next 4 days of hospitalization until dextrose infusion was successfully discontinued while maintaining euglycemia. He was discharged on diazoxide (12 mg/kg/day), chlorothiazide (10 mg/kg/day), and rhGH (0.27 mg/kg/week). His repeat echocardiogram prior to discharge remained normal. Within a week after hospital discharge, he was having persistent hyperglycemia in the 200–300 mg/dL (11.1–16.65 mmol/L) range, so diazoxide was decreased to 5 mg/kg/day.

He followed up in the endocrinology outpatient clinic 1 month after discharge. His BGs were 55–122 mg/dL (3.05–6.78 mmol/L). Diazoxide dose was increased to 7 mg/kg/day. He continued chlorothiazide and rhGH. Given the presentation of congenital hyperinsulinism, targeted next generation sequencing (NGS) with deletion/duplication analysis for 24 genes (*ABCC8*, *AKT2*, *CACNA1D*, *CREBBP*, *EP300*, *FOXA2*, *GCK*, *GLUD1*, *GPC3*, *HADH*, *HNF1A*, *HNF4A*, *INSR*, *KCNJ11*, *KDM6A*, *KMT2D*, *MAFA*, *MPI*, *NSD1*, *PGM1*, *PMM2*, *SLC16A1*, *TRMT10A*, and *UCP2*), which account for a significant portion of affected individuals [7, 8], was performed. The sequencing analysis uncovered a variant of uncertain significance in *TRMT10A* (NM_152292.4), c.1A > T (p.Met1?). Copy number assessment by NGS uncovered a pathogenic ∼5.8 Mb loss near the 5′ UTR of *FOXA2*, corresponding to 20p11.22-p11.21 (chr20: 16662148–22464994) × 1 and included 34 protein-coding genes. Due to the reported association of 20p11 deletions with gastrointestinal and genitourinary abnormalities, an ultrasound of the abdomen was performed and was normal. He had ophthalmological evaluation which showed normal bilateral optic nerves.

The patient was followed in the outpatient endocrinology clinic every 3-4 months. His medications were dose adjusted for his weight gain. His antiseizure medication was withdrawn without recurrence of seizures. Follow-up at 16 months of age and 1 year after diagnosis showed BGs were 54–139 mg/dL (3–7.72 mmol/L). His diazoxide was adjusted to 8-9 mg/kg/day. He continued to be on chlorothiazide 10-11 mg/kg/day and rhGH equivalent to 0.26-0.27 mg/kg/week. He had a repeat pituitary hormone evaluation, which revealed neither central hypothyroidism (TSH 0.92 mcIU/mL, FT4 1.0 ng/dL) nor central adrenal insufficiency (AM cortisol 10.4 mcg/dL). His sodium remained normal, and there were no concerns for polyuria nor polydipsia. His weight was 13.5 kg (97th percentile, SD 1.98) and length was 80.8 cm (85th percentile, SD 1.02).

## 3. Discussion

Hypoglycemia is clinically defined as a glucose concentration low enough to cause neurogenic and neuroglycopenic symptoms. Neurogenic symptoms are the result of sympathetic activity leading to palpitations, tremors, or sweating [9]. Neuroglycopenic symptoms are the result of deficient glucose supply to the brain leading to confusion, seizures, or even coma [9]. The clinical presentation is nonspecific and can be difficult to recognize in infants and children, leading to a delayed diagnosis. Due to the associated neurological complications, thorough investigation for the underlying etiology of hypoglycemia is essential. Etiologies of hypoglycemia presenting in infants include hyperinsulinism, growth hormone deficiency, cortisol deficiency, and inborn errors of metabolism [9]. The critical sample is obtained during a hypoglycemic event as a first-line screening for many of these etiologies [9]. A glucagon test should be performed following the critical sample to further evaluate hyperinsulinism, which is supported by an increase in BG of 30 mg/dL (1.67 mmol/L) or higher [10]. Alternatively, a poor response to glucagon may be seen in glycogen storage diseases.

Our patient's critical sample was consistent with hyperinsulinism, which is a cause of severe hypoglycemia due to increased insulin secretion from the pancreatic beta cells. It is diagnosed by detectable insulin and C-peptide levels, low beta-hydroxybutyrate, and low free fatty acids in the setting of hypoglycemia [11]. Our patient's glucose response to glucagon was lower than expected for hyperinsulinism, which may be explained by the simultaneous presence of growth hormone deficiency. In addition, in one of the original studies of glucagon as an aid in the diagnosis of hyperinsulinism [10], the authors concluded that occasionally a blunted glucose response may be seen. Other clinical features suggesting hyperinsulinism that were seen in our patient include a history of being large for gestational age as well as the infant needing a high GIR to maintain euglycemia [11].

Growth hormone deficiency in a young infant most commonly presents with hypoglycemia, prolonged jaundice, and/or microphallus [12]. Growth in infancy is mainly controlled by nutrition and genetic factors, so growth delay is not commonly seen in growth hormone deficiency. In isolated growth hormone deficiency in pediatrics, two failed GH stimulation test agents are needed to make a diagnosis [12], and in this case, our patient failed both arginine and glucagon. In addition, his subsequent genetic diagnosis is associated with growth hormone deficiency. Patient was started on growth hormone therapy but had incomplete resolution of hypoglycemia. After his baseline echocardiogram returned as normal, diazoxide and chlorothiazide were added to his treatment regimen. Diazoxide is the first-line therapy for hyperinsulinism. Due to the risk of fluid retention and pulmonary hypertension, it is recommended to concomitantly start a thiazide diuretic [13]. He responded to a diazoxide dose of 12 mg/kg/day. He was discharged home after maintaining euglycemia (BG > 70 mg/dL or>3.89 mmol/L). Our patient subsequently had hyperglycemia so the diazoxide dose was reduced. Diazoxide has a long half-life and can take up to 5 days to reach a steady state. The pharmacokinetics of diazoxide should be noted during titration of the dose, and an assessment of response is recommended after at least 5 days of therapy [13].

**Figure 1:**
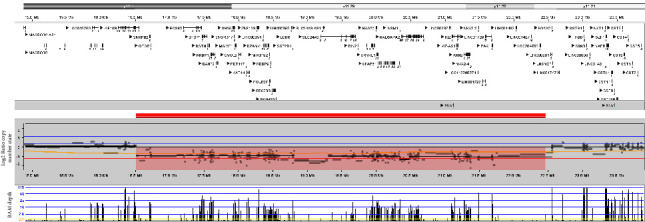
Copy number analysis by NGS detected a 5.8 Mb loss of 20p11.22p11.21, marked in red. The deletion breakpoints were chr20: 16662148–22464994 in genome build GRCh38.

Genetic testing was ordered due to multiple issues including simultaneous presence of growth hormone deficiency and hyperinsulinism. The patient's genetic testing showed a 20p11.22p11.21 deletion, which explained the simultaneous presence of growth hormone deficiency and hyperinsulinism as causes of hypoglycemia. Overlapping deletions involving this region (Figure 1) are associated with hypoglycemia due to growth hormone deficiency along with other developmental features and are de novo in origin and absent from control population datasets [1–6]. Although the causative gene within this region is unclear, there are multiple candidate genes such as *PCSK2*, which is constrained for loss of function variation in the general population and encodes a neural and endocrine proteolytic convertase involved in processing neuropeptides and hormone precursors including glucagon in pancreatic islet *α*-cells. Rare variation in *PCSK2* has been associated with type II diabetes, autism, and intellectual disability (PMID: 17618154, 3008852, and 25363768). In addition, homozygous null mice displayed a range of phenotypes including impaired processing of insulin, glucagon, and somatostatin (MGI ID: 1857445). Interestingly, the detected deletion did not include any copy number changes for the coding region or splice junctions of the genes on the targeted panel, but the proximal break point suggests that the regulatory region of *FOXA2* may be disrupted. Although forkhead box A2 (*FOXA2*) is not a disease associated gene, it encodes a transcription factor required for the formation of midline structures including the pituitary gland and the pancreas [3, 4]. Hence, the mutation in *FOXA2* is thought to play a role in panhypopituitarism and abnormal insulin secretion [1, 2]. *FOXA2* regulates the expression of genes involved in the development of the pituitary gland and pituitary hormone secretion including *SHH*, *GLI2*, and *NKX2-2* [1, 2]. *FOXA2* has also been shown to control the genes involved in insulin secretion in the pancreatic *β* cells, including *ABCC8* and *KCNJ11* [1, 2]. Interestingly, most cases of *ABCC8* and *KCNJ11* mutations are nonresponsive to diazoxide, but the positive response to diazoxide in *FOXA2* mutation is thought to be due to incomplete reduced gene expression [1, 2]. Other features of 20p11 deletions include cognitive delay, autistic behaviors, seizures unrelated to hypoglycemia, craniofacial dysmorphisms, and gastrointestinal and genitourinary anomalies [3–6]. A review of the literature indicated that the most common brain MRI abnormalities include absent and/or hypoplastic anterior pituitary, absent and/or ectopic posterior pituitary, and interrupted pituitary stalk [1–5]. To our knowledge, this is the first case of 20p11 deletion to have reported a normal brain MRI.

This case adds to the 3 cases [1–3] of 20p11 deletion found in the literature that describe patients who presented with hypoglycemia, later diagnosed to have a combination of growth hormone deficiency and hyperinsulinism responsive to diazoxide. All 3 cases report persistent hypoglycemia shortly after birth that required treatment. It is possible that our patient had hypoglycemia since birth as well. Symptoms of hypoglycemia are difficult to recognize in infants, which can lead to a delayed diagnosis. In our case, BG was not checked on initial presentation. This case is a reminder that seizure is a presentation of severe hypoglycemia, so a BG should be checked mandatorily in any child presenting with a seizure. All 3 cases, as well as our patient, were responsive to diazoxide treatment; maximum dose was reported at 15 mg/kg/day [1]. Hyperinsulinism in 20p11 deletion may be persistent as reported in 2 of the cases [1, 3] where patients still needed diazoxide at age 5 to 6 years. Two of the patients [2, 3] had other features of hypopituitarism, including both central hypothyroidism and central adrenal insufficiency that were diagnosed at the time of the hypoglycemia evaluation.

## 4. Conclusion

20p11 deletion is associated with hypopituitarism and hyperinsulinism that can present as hypoglycemia in infancy. It is crucial to recognize that a seizure in infancy and childhood can be caused by significant hypoglycemia and, therefore, an appropriate evaluation of blood glucose should be performed in all infants presenting with new onset seizures. This unique case shows that two pathological conditions causing hypoglycemia can coexist, so the differential diagnosis for hypoglycemia should remain broad. In addition, if hypoglycemia continues despite treatment, secondary causes should be explored.

## Figures and Tables

**Table 1:** Results of critical sample.

Test	Result	Normal range during hypoglycemia
Serum glucose	39 mg/dL^a^	<50 mg/dL^f^
Insulin	3.2 mcIU/mL^b^	<2 mcIU/mL^g^
C-peptide	0.6 ng/mL^c^	<0.6 ng/mL^h^
Beta-hydroxybutyrate quant	145.6 mcmol/L	>600 mcmol/L
Human growth hormone	4.2 ng/mL^d^	>10 ng/mL^i^
Cortisol	15.7 mcg/dL^e^	>18 mcg/dL^j^
Free fatty acids	0.49 mmol/L	<0.5 mmol/L
Lactic acid	1.0 mmol/L	0.7 to 2.1 mmol/L
Ammonia	<9 mcmol/L	<62 mcmol/L

SI units: ^a^2.17 mmol/L, ^b^22.24 pmol/L, ^c^0.198 nmol/L, ^d^4.2 mcg/L, ^e^433.32 nmol/L, ^f^<2.78 mmol/L, ^g^<13.9 pmol/L, ^h^<0.198 nmol/L, ^i^>10 mcg/L, and ^j^>496.8 nmol/L.

**Table 2:** Results of glucagon test.

Test	Result (mg/dL)
Glucose 0 min	35^a^
Glucose 10 min	48^b^
Glucose 20 min	57^c^
Glucose 30 min	61^d^
Glucose 40 min	51^e^

SI units: ^a^1.94, ^b^2.67, ^c^3.17, ^d^3.39, and ^e^2.83 mmol/L.

**Table 3:** Results of growth hormone stimulation test.

Time	Growth hormone (ng/mL or mcg/L)	Serum glucose (mg/dL)
0 min (time 10:49)	1.8	48^a^

*Arginine (0.5 mg/kg* *=* *3.3 mg)*		
30 min (11:22)	2.5	59^b^
60 min (11:57)	1.9	46^c^
90 min (12:27)	3.8	45^d^

*Glucagon (0.03 mg/kg* *=* *0.2 mg)*		
120 (12:57)	5.5	59^e^
150 (13:25)	6.6	55^f^
180 (13:58)	4.7	55^g^

SI units: ^a^2.67, ^b^3.27, ^c^2.55, ^d^2.50, ^e^3.27, ^f^3.05, and ^g^3.05 mmol/L.

## Data Availability

The data used to support the findings of this study are included within the article.
